# Preliminary analysis reveals links between visual attention, sleep duration, and neural recruitment in Alzheimer's disease

**DOI:** 10.1002/alz.093836

**Published:** 2025-01-09

**Authors:** Maggie P Rempe, Seth D Springer, Grant M Garrison, Ryan J Glesinger, Hannah J Okelberry, Aubrie J Petts, Christine M Embury, Tony W Wilson

**Affiliations:** ^1^ Boys Town National Research Hospital, Omaha, NE USA; ^2^ University of Nebraska Medical Center, Omaha, NE USA

## Abstract

**Background:**

Sleep disturbances have been shown to relate to the development of Alzheimer’s disease (AD) and its associated cognitive deficits, including visual attention function. Here, we investigated the potential interactive and additive effects of sleep disruption and AD on visual attention performance and its underlying neural mechanisms.

**Method:**

Preliminary analysis included 33 participants, 10 biomarker‐positive patients on the AD spectrum (mild cognitive impairment (MCI) and AD; 7 females; Mage: 70.24) and 23 cognitively‐healthy older adults (17 females; Mage: 67.47). Participants completed a visual search task during magnetoencephalography (MEG). Participants wore an Actigraph device on their non‐dominant wrist for a two‐week period. Actigraph data were cleaned using sleep diaries and analyzed using the Cole‐Kripke algorithm. Total sleep time (TST) was calculated for each night of sleep and averaged across all nights of sleep collected. The MEG data were transformed into the time‐frequency domain and significant task‐related oscillatory responses were source‐imaged.

**Result:**

Behavioral analysis of the visual search task revealed a significant conditional effect (p < .001) and a significant group by TST interaction (p < .001) on RTs, revealing overall longer RTs in conjunctive trials and longer RTs in patients on the AD spectrum with shorter TST. Regarding the MEG data, we observed robust neural responses in theta, alpha, and beta frequency bands. Analysis of source‐imaged neural responses in the alpha range revealed significant conditional differences in the right posterior temporal lobe (p < .005), such that stronger responses were found in the conjunctive condition relative to the feature condition. A significant group‐by‐condition interaction was found in the right temporoparietal junction (TPJ; p < .005), revealing that patients on the AD spectrum had weaker responses during the conjunctive condition compared to controls. Finally, a group by TST interaction on conditional differences in alpha power (p < .005) in right occipital and cerebellar regions revealed that the relationship between condition‐wise neural recruitment in these regions and TST differed as a function of AD diagnosis.

**Conclusion:**

These preliminary findings suggest key relationships between total sleep time, visual attention behavioral performance and neural recruitment, and how these relationships may differ in AD spectrum conditions.

